# The Application of Non-Parametric Count Models for the Modeling of Female’s Accident Rates in Hamadan Province from 2009 to 2016

**Published:** 2020-04

**Authors:** Mostafa EGHBALIAN, Abbas MOGHIMBEIGI, Marzieh MAHMOODI, Iraj MOHAMADFAM, Razieh Sadat MIRMOEINI

**Affiliations:** 1. Department of Biostatistics, School of Public Health, Hamadan University of Medical Sciences, Hamadan, Iran; 2. Modeling of Noncommunicable Disease Research Center, Hamadan University of Medical Sciences, Hamadan, Iran; 3. Department of Biostatistics, Faculty of Health and Nutrition, Bushehr University of Medical Sciences, Bushehr, Iran; 4. Department of Occupational Health, School of Public Health, Hamadan University of Medical Sciences, Hamadan, Iran; 5. Disease Control & Prevention Center, Deputy of Health Services, Hamadan University of Medical Sciences, Hamadan, Iran

**Keywords:** Poisson, Negative binomial, Semiparametric mixed model, Accident, Iran

## Abstract

**Background:**

Accidents were just one of the general health problems. According to WHO forecasts (2013), deaths from road accidents will become the fifth-highest cause of death in the world by 2030. Therefore, we have attempted the application of non-parametric count models for modeling female’s accident rates.

**Methods:**

All accidents in Hamadan Province, western Iran are referred to as one of the emergency centers located in the hospitals. Data regarding the accidents were obtained from 21 emergency centers across Hamadan for the period 2009–2016. To assess the trend and pattern of the accidents, the Generalized Additive Model for the accident rate has been utilized.

**Results:**

The Mean±SD age of the females in study was 31.23±12.88 yr old. For each of the three kinds of road accidents (car accidents, motor accidents and pedestrian crashes), the accident rates in the “residential urban” areas are lesser than in the “non-residential” area (*P*=<0.001) and in “public and sports grounds” and “great roads, avenues and streets” are more than in “others”. For the three kinds of accidents, the functional effect in the monthly trend of the accidents was signification (*P*=<0.001).

**Conclusion:**

The rates for all three kinds of accidents decreased. The increase in accident rates from the beginning of 2014 to Mar 2016 maybe due to the generalization of insurances in Iran and the increase in the number of accident victims being referred to the hospitals, which was the same with the results of other studies.

## Introduction

According to WHO, an accident is an unexpected, unplanned, and non-historical event that usually results in injuries, deaths, or special injuries that are unwanted and recognizable. Studies on accident prevention constitute an important part of applied research, and they can be important to the formulation of the country plans (1). Universal time (daily) injuries and accidents will become the third most critical cause of death after diseases and infections in Asia, especially in Central Asia (1). At the beginning of the 21st century, road accidents were just one of the general health problems. According to WHO, road accidents caused 1.3 million deaths and about 20–50 million injuries in 2013 (2). In 2004, road accidents were the ninth highest cause of death in the world. According to WHO forecasts (2013), deaths from road accidents will become the fifth-highest cause of death in the world by 2030. In addition, the highest incidence rate of road accidents is among people aged between 15 to 29 yr—this shows that the problem is quite serious (2).

Injuries and deaths resulting from motorcycle accidents are quite common; they account for about 1.24 million deaths and around 20–50 million injuries across the world every year (3). The safety of motorcyclists is a major issue in developing countries. In some of these countries, accidents caused by motorcycles comprise 60% of the total road accidents in a specific year (4). The number of motorcycle passenger fatalities totaled 332 or seven percent of all motorcycle fatalities including both riders and passengers. Based upon a ten-year average from 1996 to 2007, women motorcycle passengers account for ninety percent of women motorcycle injuries (5). Motor vehicle traffic crashes accounted for approximately 33% in 0–4 yr, 50% in 5–14 yr and 75% in 15–19 yr age of all unintentional injury deaths (6). In Sweden, approximately 12,000 people are hospitalized annually due to motorcycle accidents (3). Pedestrians are the most vulnerable victims of traffic accidents. In 2012, about 21% and 10% of the total fatal accidents in an undisclosed union and America respectively were among pedestrians (7). In 2013, 13,000 pedestrians were involved in road accidents in Taiwan that resulted in 415 deaths. In addition, about 13% of the total deaths were due to traffic accidents in Taiwan (8). In Spain, the formal statistics for the year 2011 reveals that about 10,865 pedestrians were injured and 380 people killed due to road accidents. Here 18% of the total mortality could be attributed to traffic accidents (9).

In Iran, the mortality rate due to road accidents is a critical concern. In recent years, the government has been more aware of this. In Iran, about 22,918 people were killed in 2008 because of road accidents. According to this report, Iran has most mortality among 22 countries in eastern Mediterranean, Iran is accounted for 30% of the total mortality. Iran is the third most crowded country in the Middle East and 32% of the region’s automobiles are in Iran. The mortality rate due to road accidents across the world and in the Middle East are 18.8% and 32.2% per 100,000 people respectively. In Iran, it is 35.8% per 100,000 people (10).

In similar studies that have been used to model road accident data, parametric models such as Poisson regression and negative binomial regression have been used; the negative binomial regression was used on the data on road accidents that occurred in Singapore during 1992–1999 (11). The semi-parametric negative binomial model was used for the data regarding car crashes, medium daily traffic, and numerically designed data from 1995–1999 (12). The generalized additive model (GAM) has been used to investigate the trend in data with counting responses such as count of rotten, felled, and put out teeth and the weather and emergency call data. The general additive model was used on the data about cesarean sections performed in Hamadan’s hospitals (13). In another study, a zero-inflated multilevel semi-parametric model used with overdispersion DMFT (count of rotten, felled, and put out teeth) data (14–15). Feng Chen et al. used the general additive linear model on the weather and emergency call data in Hong Kong(16). Because the accident rates have changed over time with an unknown function, the nonparametric GAMs are more appropriate than the parametric models to identify the trend of female’s accident rates and effective factors.

Therefore, in this study, we attempted to use the non-parametric count models for modeling female accident rates in Hamadan province during 2009–2016.

## Materials and Methods

All accidents in Hamadan province are referred to one of the emergency centers located in the hospitals. In this retrospective study, the data regarding the accidents were obtained from 21 Hamadan Province hospital emergency centers for the period between Mar 2009 and Mar 2016. Hamadan Province is located to the west of Iran. According to the scale of 2011, the population of Hamadan Province was 12, and according to the census of 2016, the population of Hamadan Province was 10. Based on the 2011 census, the population of Hamadan is 1,758,268 and Based on the 2016 census, the population of Hamadan is 1,738,234 (17).

To assess the trend and pattern of the accidents, the GAM for the accident rate has been utilized. The response variable in this study is the rate of admission of female accident victims in each of the hospitals.

The dataset also included the variables affecting the accidents: age (The average age of people referring to a hospital in a month), the type of venue of the accident (“public and sports grounds”, “great road, avenue, and street” and “workplace, school, and educational place,” and “others”), and the type of the accident areas (“urban residential area”, “rural residential area”, and “non-residential area”).

In statistics, an additive model is a regression model in which the response variable depends linearly on the unknown smooth functions of certain predictor variables. The Poisson additive model is a GAM for counting data, defined as follows:

[1] $$\log(\mu_{i})={\text{Z}}_{\text{i}}\beta +f_{1}(x_{1i})+f_{2}(x_{2i})+\ldots +f_{p}(x_{pi})$$

*Y*_*i*_ ∼ *Poisson* (*μ*_*i*_)

*E*(*Y*_*i*_) = *μi*) And *Var* (*Y*_*i*_) = *μi*)

Where Y_i_ is the outcome variable for subject i, **Z**_i_ is a row of the design matrix for any parametric covariates and **β** is the regression coefficients vector. The functions *f*_*j*_ are smooth functions of the nonparametric covariates *x*_*k*_that are to be estimated.

In the Poisson additive model for calculating the rate, an offset should be added to the right-hand side of the equation, as shown in Eq. 1. The offset for the data for this study is defined as the natural logarithm of the unit population.

The negative binomial additive model is also appropriate for generating count data models. The negative binomial model is usually used when overdispersion exists. Empirically, it has often been found that count data exhibits overdispersion when the variance is larger than the mean.

In the negative binomial additive model, the response variable Y_i_ has a negative binomial distribution. The mean of these distribution models such as [1] with mean and variance as below:

$$\text{E}({\text{Y}}_{\text{i}})=\mu_{i}\;\text{And}\;Var(Y_{i})=\left(1+\frac{\mu_{i}}{r}\right)\mu_{i}$$

Where r^−1^ is called the overdispersion parameter.

Mixed models are especially useful in longitudinal studies where repeated measurements are made on the same statistical units, or in settings where measurements are made on clusters of related statistical units. Owing to their advantage in handling missing values, mixed models are often preferred over traditional approaches, such as the repeated measures model (18).

In this study, the data was gathered on a monthly basis (from Mar 2009 to Mar 2016); therefore, the additive mixed model for the rate is as follows:

$$\log(\mu_{i})={\text{Z}}_{\text{i}}\beta +f_{1}(x_{1})+f_{2}(x_{2})+\ldots +f_{p}(x_{p})+offset+v_{i}$$

Where *v*_*i*_ is considered as the hospital-specific effects (random effects) whose values have a normal distribution with the mean 0 and variance *σ*_*v*_.

In this study, we have employed a generalized additive mixed model (GAMM) under the Poisson and the negative binomial distribution for the count data of the accidents. The functions and parameters of the model are estimated by using the packages “gamm4”, “mgcv” and “lme4” in the software R (ver. 3.2.2).

### Ethical approval

The data from this research was collected from Hamadan Deputy of Health Services. Code of ethics for collecting data is IR.UMSHA.REC.1396.57.

## Results

The total number of females involved in road accidents in three kinds of accident were 18,525 (car accidents: 12,910 (69.69%), motor accidents: 2,122 (11.45%), and pedestrian crashes: 3,493 (18.86%)) from Mar 2009 till Mar 2016. The mean age of the females in this study was 31.23 ± 12.88 yr old.

At first, we applied the statistical models (Poisson model, mixed Poisson model, negative binomial model, and mixed negative binomial model) and then determined which model to use according to Akaike Information Criteria (AIC). For car accidents, the AIC=1662.261; for motor vehicles accident, the AIC=2275.830. The mixed negative binomial model had the least AIC, while the AIC was 2093.160for pedestrian crashes. The mixed Poisson model showed the least AIC. According to Table 1, the rate (**exp(β)**) of accident areas “Urban residential” to “non-residential area” was equal to 0.967 (*P*=<0.001) and the rate of accident place “public and sports grounds” to “Other” was equal to 1.092 (*P*=<0.001) and the rate (**exp(β)** of “Great road, avenue, and street” to “Other” was equal to 1.878(*P*=<0.001) were significant. In this estimate, the trend of the non-parametric model during the time (month) was 2 degrees (edf=2.518) and showed a significant relation (*P*=0.005).

**Table 1:** Parameter estimates and standard errors for mixed negative binomial regression models for car accidents

***Parameter***	***Estimate β***	***Standard error***	***Statistical t***	**P*-value***
Intercept	−9.913	0.174	−56.951	<0.001
Accident area				
Rural residential	0.003	0.007	0.482	0.630
Urban residential non-residential area	−0.034	0.003	−11.585	<0.001
Accident place				
Public and sports grounds	0.088	0.006	14.754	<0.001
Great road, avenue and street	0.630	0.003	20.943	<0.001
Work place, school and educational place	−0.084	0.227	−0.368	0.713
Other				
Non-parametric part				
		DF estimate	Statistical F	*P*-value
S(Month)		2.518	4.875	0.005
	AIC	Adj R^2^	Log likelihood	
	1662.261	−16100	−821.130	

**Fig. 1:**
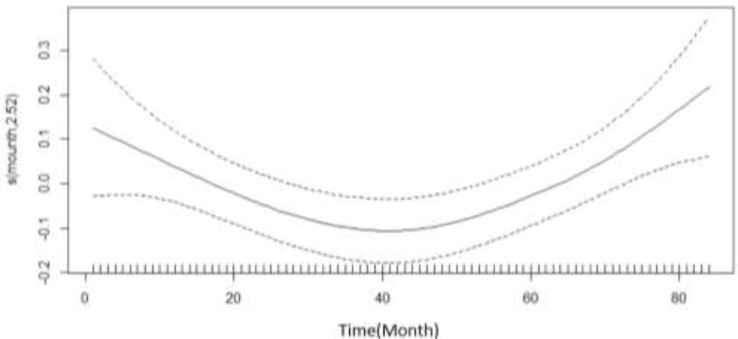
Regression spline functions depicting an estimate of the monthly trend of cars accident

Figure 1 demonstrates the monthly trend of car accidents. The plot shows the change of the function of duration with regards to duration and confident interval. From Mar 2009 to Sep 2010, the rate of accidents increased at a decreasing rate. Between September 2010 and Sep 2014, the rate of accidents decreased at a first with increasing rate and then with decreasing rate, and then, between Sep 2014 and Mar 2016, the rate of accidents increased at an increasing rate.

According to the results demonstrated in Table 2, the rate (**exp(β)**) of accident areas “Rural residential” to “non-residential area” was equal to 1.284 (*P*=<0.001) and the rate (**exp(β)**) of “Urban residential” to “non-residential area” was equal to 0.859 (*P*=<0.001), the rate (**exp(β)**) of accident place” public and sports grounds” to “Other” was equal to 1.459 (*P*=<0.001) and the rate (**exp(β)**) of “Great road, avenue and street” to “Other” was equal to 1.292 (*P*=<0.001) were significant. In this estimate, the trend of the non-parametric model during the time (month) was 3degrees (edf=2.959), and showed a significant relation (*P*=<0.001).

**Table 2:** Parameter estimates and standard errors for mixed negative binomial regression models for motor accidents

***Parameter***	***Estimate β***	***Standard error***	***Statistical t***	**P-*value***
Intercept	−11.718	0.107	−109.168	<0.001
Accident area				
Rural residential	0.250	0.050	5.040	<0.001
Urban residential non-residential area	−0.152	0.031	−4.967	<0.001
Accident place				
Public and sports grounds	0.378	0.049	7.683	<0.001
Great road, avenue and street	0.256	0.037	7.000	<0.001
Work place, school and educational place	0.332	0.338	0.983	0.326
Other				
Non-parametric part				
		DF estimate	Statistical F	*P*-value
S(Month)		2.959	17.300	<0.001
	AIC	Adj R^2^	Log likelihood	
	2275.830	−3.1e+09	−1127.915	

**Fig. 2:**
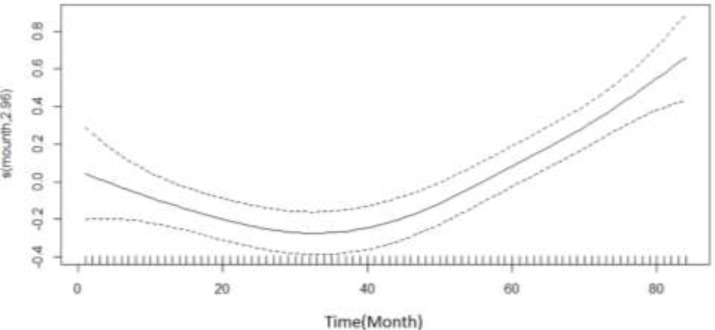
Regression spline functions depicting an estimate of the monthly trend of motors accident

Figure 2 demonstrates the monthly trend of car accidents. From beginning of study to Sep 2013, the rate of accidents decreased at a first within creasing rate and then with decreasing rate between Sep 2013 and Mar 2016, the rate of accidents increased at an increasing rate.

According to the results demonstrated in Table 3, the rate (**exp(β)**) of accident areas “Rural residential” to “non-residential area” was equal to 1.074 (*P*=<0.001) and the rate (**exp(β)**) of “Urban residential” to “non-residential area” was equal to 0.974 (*P*=0.002), the rate (**exp(β)**) of accident place “public and sports grounds” to “Other” was equal to 1.075 (*P*=<0.001), the rate (**exp(β)**) of “Great road, avenue and street” to “Other” was equal to 1.067 (*P*=<0.001) and the rate (**exp(β)**) of “workplace, school, and educational place” to “Other” was equal to 1.130 (*P* =<0.001) were significant. In this estimate, the trend of the non-parametric model during the time (month) was 3 degrees (df=2.944), and showed a significant relation (*P*=<0.001).

**Table 3:** Parameter estimates and standard errors for mixed poison regression models for pedestrian accidents

***Parameter***	***Estimate β***	***Standard error***	***Statistical t***	**P *-value***
Intercept	−11.031	0.253	−43.527	<0.001
Accident area				
Rural residential	0.071	0.014	5.064	<0.001
Urban residential non-residential area	−0.026	0.008	−3.084	0.002
Accident place				
Public and sports grounds	0.073	0.013	5.758	<0.001
Great road, avenue and street	0.065	0.009	7.408	<0.001
Work place, school and educational place	0.122	0.032	3.821	<0.001
Other				
None-parametric part				
		DF estimate	Statistical F	*P*-value
S(Month)		2.944	22.83	<0.001
	AIC	Adj R^2^	Log likelihood	
	2093.160	0.861	−1036.580	

**Fig. 3:**
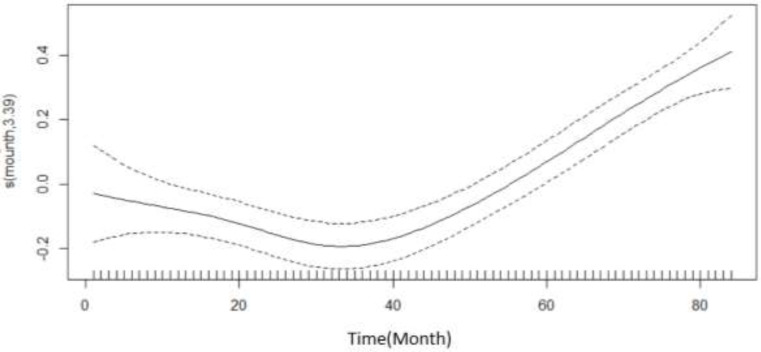
Regression spline functions depicting an estimate of the monthly trend of pedestrian crashes

Figure 3 demonstrates the monthly trend of car accidents. From beginning of study to Sep 2013, the rate of accidents decreased at a first with increasing rate and then with decreasing rate, between Sep 2013 and Mar 2016, the rate of accidents increased at an increasing rate.

## Discussion

In this study, we applied the GAMs of Poisson and negative binomial regression to examine the main factors responsible for traffic accidents among females in Iran’s Hamadan Province. GAMs involve clustered or repeated measurements of the same subject over time with a flexible function of time. Since the additive model does not require a specific functional form, the GAM is often preferred over more traditional approaches, such as the General Linear Model (GLM) (19).

From this study, the rates of accidents were as follows: car accident=69.69%; pedestrian accidents=18.86%, and motorcycle accidents=11.45%. In this study, it was unclear whether women were themselves, drivers or occupants. The data was only a breakdown of the car rider, motorcyclist, or pedestrian accident. The highest accident rates were for motorcycle accidents at 55.7%, followed by car accidents at 18.8%, and last were pedestrian accidents at 18.8% (20).

The mean age of the subjects in this study was about 31 yr old. According to the WHO report, about 60% of all deaths caused due to road accidents are related to the people aged between 15–44 yr (21). Therefore, this productive class of society should be directed by the authorities toward attending the accident reduction programs.

For each of the three kinds of road accidents (car accidents, motor accidents, and pedestrian crashes), the accident rates in the “residential urban” areas are lesser than in the “non-residential” area. For motor and pedestrian accidents, the rates in “residential rural” areas are more than those in the “non-residential” areas. For each of the three kinds of road accidents, the rates in “public and sports grounds” and “great roads, avenues and streets” are more than in “others” and for pedestrian accidents, “workplaces, schools and educational places” are more than “others”.

In this investigation, for the three kinds of accidents, the functional effect in the monthly trend of the accidents was significant. Between Mar 2009 and Sep 2010, the rate of car accidents increased with a decreasing rate. Between Sep 2014 and Mar 2016, the rate of car accidents again increased. In addition, at the beginning of 2014, the rate of motor vehicles and pedestrian accidents increased at first and then decreased.

According to the accident reports from 2009 to 2014, the rates for all three kinds of accidents decreased. This finding is in line with the results of another study (22). This can be attributed to certain factors such as changes in the traffic laws since the police have grown more stringent in recent years. The increase in accident rates from the beginning of 2014 to Mar 2016 could be due to the generalization of insurances in Iran and the increase in the number of accident victims being referred to the hospitals.

the time series method was used to investigate automobile accidents in the province of Hamadan from 2010 to 2016 in Fars City. According to the results of this study, the mortality followed an ascending trend in this study. Among male patients, mortality trend was relatively fixed, but showed an increasing slope in some months of the year. However, this trend increased continuously in females (23). The author sees increased inclination toward women’s work and activities in society and the need for personal cars due to the increasing incidence of driving in women (24, 25).

The specific involvement of the female population in road accidents has not been extensively studied in Iran as well as in other countries. Hence, we could not perform a significant comparison of our results with other studies.

Rural children and youth in Alberta, Canada were three times more likely to be admitted to hospital following a motor vehicle crash from 1997 to 2002. These increases in risk (rural versus urban) were consistent across age groups and sex (6).

An increased risk of fatal and severe motor vehicle crash injuries among children and youth in rural (versus urban) environments has been previously documented (26). A Nevada study examining severe motor vehicle crash injuries (27) showed that rates among children (0–14 yr) were three times higher in rural areas, compared with urban areas. In contrast, however, to the two- to three-fold increased risk of fatal crash involvement in rural (versus urban) areas reported by others (26) we observed a five-fold increase in risk.

The rural drivers, compared with urban drivers, spend more time on the road and drive longer distances (28). In an ecologic analysis of United States Federal Highway Administration data, estimates of vehicle miles traveled per capita (in thousands) ranged from 10.0 to 52.5 in rural areas (median of 15.0) and from 5.3 to 12.2 in urban areas (median of 8.0) (29).

The logistical difficulties and financial constraints faced by police agencies responsible for enforcing traffic laws on low volume rural roads may contribute to these urban-rural differences in unsafe driving behaviors. For example, despite a lower self-reported prevalence of speeding in urban areas, urban drivers in Australia were more likely than rural drivers (20% versus 15%) to have received a citation for speeding in the previous two years (6).

In Gillan, northern Iran major accidents had occurred in the urban areas (50.3%) (30). A survey was conducted on the country’s motorcycle accidents in 2013. In this study, the major accidents had occurred in the city (54.80%), while the highest rate of motor vehicle accidents was found in summer (64.30%). However, motor vehicles accidents were the lowest in winter (19.45%) (31). In Fars, the mortality rate was investigated between the years 2004 and 2010. They used linear regression and chi-scour for data analysis by considering the factor that the mortality rates among females has increased (32).

Among the limitations of this study, some of these accidents were for women traveled to the cities of Hamadan, and these women were considered as citizens of Hamadan, or the Hamadan women who crashed in other cities were not included in the study. This study was conducted only in women of Hamadan province. More comprehensive research could be conducted throughout the country to determine the traffic accidents in women throughout the country.

## Conclusion

As discussed above, in the years after 2014, the incidence of accidents occurred in all three events for women in Hamadan Province that may be because in recent years the number of certificates for women has increased, which should be addressed to the authorities. Moreover, for data on driving accidents, the subjects are correlated with each individual and the results indicate that the model is appropriate.

## Ethical considerations

Ethical issues (Including plagiarism, informed consent, misconduct, data fabrication and/or falsification, double publication and/or submission, redundancy, etc.) have been completely observed by the authors.
